# The Role of Catheter Ablation in Military Personnel with Atrial Fibrillation

**DOI:** 10.1093/milmed/usz126

**Published:** 2019-06-11

**Authors:** Kelvin N V Bush, Gregg G Gerasimon

**Affiliations:** Division of Cardiology, San Antonio Military Medical Center, 3551 Roger Brooke Dr., San Antonio, TX

The lifetime risk of developing atrial fibrillation (AF) increases with age and is approximately 25% for both men and women.^1^ Despite AF having strong associations with increasing age, this very common arrhythmia occurs in the population under 60 years of age at less than 1%. The military population with AF merits particular attention given the increased morbidity and subsequent occupational impact. Current guidelines and expert discussions address medical and catheter based therapies for the general population, however, there is a paucity of literature supporting a standard AF management in the young servicemember. This commentary discusses the role of catheter ablation for atrial fibrillation in the servicemember.

## MILITARY EXERCISE AND THE ASSOCIATION WITH AF

Athletic individuals and many military members participate in higher intensity exercise and these high-intensity lifestyles have been linked to an increased risk of developing AF.^2^ This demographic group is a significant contrast to traditionally older patients with structural heart disease and comorbidities promoting atrial fibrosis and chamber dilation. Those athletes with regular and higher-intensity exercise have frequent variations in their autonomic tone: high vagal tone at rest and high sympathetic activation with exercise. It is thought that these frequent and dramatic swings in autonomic tone may play a role in predisposing a young athlete to AF. Turagam et al. showed an increased AF incidence in a population of young athletic men with high resting vagal tone and low baseline heart rates.^3^ In light of these physiologic observations, there exists a subtype of AF known as “vagally-mediated AF,” in which AF occurs in athletes when asleep or when vagal tone is most pronounced (eating, alcohol consumption, relaxation following stress or exercise, post intercourse).^4^ In addition to the autonomic tone fluctuations observed in young athletes, researchers have observed chronic endurance exercise promoting left atrial fibrosis and remodeling. It is the complex interaction between the sympathetic-parasympathetic tone, inflammatory milieu, and fibrotic remodeling of the atria that postulates the promotion of AF initiation and maintenance in the young athlete and military population.

## MEDICAL THERAPY FOR AF

Pharmacologic rate control agents include beta blockers, nondihydropyridine calcium channel blockers, digoxin, and some antiarrhythmics. Beta blockers have generally been the most commonly utilized rate control agent and are variably tolerated in younger patients secondary to the side effect profile of inducing fatigue, symptomatic bradycardia, erectile dysfunction and other common side effects. Beta blockers have the potential to theoretically aggravate AF symptoms in those servicemembers with vagally-mediated AF.

Rhythm control strategies entail antiarrhythmic drug therapy (AADT) and catheter ablation options. For young athletes and patients with vagally-mediated AF, guidelines refer to disopyramide, an AADT with long-acting anti-cholinergic properties as first line pharmacotherapy.^5^ The “pill in pocket” approach with flecainide can be utilized for a subset of servicemembers with paroxysmal and symptomatic AF without the burden of long-term medical therapy. Rate controlling agents and all AADTs prescribed to flyers, flight operators, divers, rangers, and other high risk occupations for AF require medical evaluation boards and medical waivers. These medical therapies with AF can limit mobility profiles and their efficacy is highly variable. Prior meta-analyses have suggested an overall efficacy of AADT at only 46% of preventing AF recurrences at 6–12 months^6^ while other meta-analyses and systemic reviews on AADT have shown significantly reduced recurrences of AF.^7^ Additionally, it is not uncommon for AADT to be discontinued because of inappropriate or intolerable side effects in clinical practice and there are mixed literature reviews reporting these rates. The medical community discussion surrounding AADT side effects and variable efficacies have driven the development of catheter ablation technology for AF.

## CATHETER ABLATION FOR SERVICEMEMBERS

**FIGURE 1.**
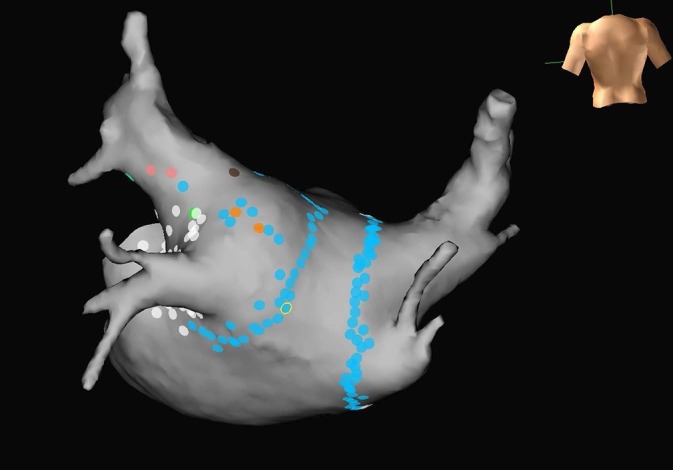
Mapped pulmonary veins with complete pulmonary vein isolation lesion sets

Since the 1990s, our increasing knowledge about the pulmonary veins and enhanced technologies have developed pulmonary vein isolation (PVI) for AF. The procedure involves obtaining vascular access via the femoral vein and then proceeding to enter the left atrium from the right atrium via a transseptal puncture. The transseptal puncture is performed under both intracardiac echocardiogram and fluoroscopic guidance. Most operators aim for a lower and more anterior transseptal puncture to allow for enhanced balloon rotation posteriorly and to reach the inferior pulmonary veins. Systemic anticoagulation is then initiated with intravenous heparin and therapeutic anticoagulation is maintained throughout the procedure in order to prevent thrombi from forming on the catheters while in the left atrium. Pulmonary vein isolation is effectively performed when all pulmonary veins are isolated using radiofrequency ablation (RFA) or cryoablation (Fig. 1). The traditional RFA method creates point-to-point lesions that encircle all four of the pulmonary veins. Cryoablation is an alternative energy source that delivers liquid nitrous oxide under pressure through a balloon to freeze the nearby and surrounding tissues. Three-dimensional electroanatomical mapping, navigation systems, and intracardiac echocardiography assist operators with orientation and localization of the pulmonary vein anatomy. The absence of pulmonary vein potentials as identified by mapping catheters inside the pulmonary veins is indicative of a successful PVI. If there are persistent vein connections to the left atrium, additional ablation should be performed. Maneuvers to provoke pulmonary vein and non-pulmonary vein triggers of AF are performed with infusions of isoproterenol and adenosine post-ablation. After PVI it is recommended that patients are maintained on systemic anticoagulation ≥ 2 months post procedure in order to prevent thromboembolism around the time of RFA.^8^ After successful PVI, symptom and ambulatory monitoring can be done to evaluate for AF recurrence months after a procedure. With PVI servicemembers can be potentially offered freedom from AF without the need for chronic medical therapy and preserved mobility eligibility. Many military personnel with lower risk positions in administration, maintenance, engineering, information technology and others could be considered by respective medical boards to maintain their occupation after PVI coupled with freedom from AF.

## COMPLICATIONS OF PULMONARY VEIN ISOLATION

The complications of PVI, though rare, include transient ischemic attack, stroke, pericardial effusion with cardiac tamponade, pericarditis, pulmonary vein stenosis, vascular complications, phrenic nerve injuries, atrial-esophageal fistulas, gastric hypomotility, iatrogenic atrial flutter, mitral valve entrapment, radiation injuries, air embolism, asymptomatic cerebral emboli, coronary artery stenosis, stiff left atrial syndrome, and death. Knowledge and awareness of these complications could potentially steer a primary provider away from considering catheter ablative therapies if one is not aware of the current and contemporary complication rates as the majority of these selected complications occur with an incidence of <2%. Six electrophysiologists in the past three years with 506 AF ablations at the San Antonio Military Medical Center report zero pericardial effusions and zero pericardial effusions requiring pericardiocentesis. The national standard for mandated reporting of pericardial effusion metrics related to AF is < 5%. When other rare procedural complications and/or deaths have occurred there are peer reviews, root cause analyses, and targeted interventions to mitigate and minimize future procedural risks as with any procedure evaluation. In response to significant complications there have been remediation and educational efforts to minimize collateral injuries by emphasizing the utilization of intracardiac echocardiography, esophageal temperature monitoring, and computed tomography to enhance individual pulmonary vein anatomy understandings. Current efforts are being made to enroll military treatment facilities into the national cardiovascular database registry for systematic and formal reporting of procedural outcomes in cardiovascular procedures.

Table I references complications and incidences reported (with full credit given) from the 2017 expert consensus statement on catheter and surgical ablations of AF on the general population.^5^ In the study conducted by Leong-Sit et al., their rates of complications correlated with increasing age. There were no major complications and only 2 vascular complications observed in the group under 45 years of age. Of the total 2,038 ablation procedures throughout all age groups, major complications and other complications occurred at 1.7% and 2.6%, respectively.^9^ Quality measures and outcomes of catheter ablation for AF in military treatment facilities are self-reported by individual electrophysiologists when counseling each servicemember.

**TABLE I.** Complications of AF Ablation and the Published Incidences in the General Population.

Complications of AF Ablation	Published Incidences
Air Embolism	<1%
Asymptomatic cerebral emboli	2–15%
Atrial esophageal fistula	0.02–0.11%
Cardiac Tamponade	0.2–0.5%
Coronary artery stenosis/occlusion	<0.1%
Death	<0.1–0.4%
Gastric hypomotility	0–17%
Mitral valve entrapment	<0.1%
Pericarditis	0–50%
Permanent phrenic nerve paralysis	0–0.4%
Pulmonary vein stenosis	<1%
Radiation injury	<0.1%
Still left atrial syndrome	<1.5%
Stroke and transient ischemic attack	0–2%
Vascular access complications	0.2–1.5%
Femoral pseudoaneurysm	
Arteriovenous fistula	
Hematoma	

## FINAL DISCUSSION FOR CATHETER ABLATION

The management of AF is centered on symptom management, preventing cardiomyopathies, and stroke prophylaxis. Current AF guidelines recommend catheter ablation in patients with AF refractory or intolerant to at least one Class I or III antiarrhythmic medication.^8^ Catheter ablation therapy for AF can be offered to patients as first line therapy after weighing the risks of the procedure and properly selecting the appropriate patients (Class IIa recommendation, Level B evidence).^8^ With the Class IIa recommendation most military electrophysiologists would recommend performing PVI in servicemembers who are highly symptomatic and in those with a moderate-high AF burden. The CABANA Trial (Catheter Ablation vs Antiarrhythmic Drug Therapy in Atrial Fibrillation) is the largest and most recent randomized, prospective trial that was designed to compare the safety and efficacy of catheter ablation to drug therapy for the treatment of patients with new-onset or untreated AF. This trial of predominately patients older than 65 years of age failed to demonstrate superiority of catheter ablation for cardiovascular outcomes (total mortality, stroke, serious bleeding, or cardiac arrest) at 5 years among patients with new-onset or untreated AF. It should be noted that very few patients in the CABANA trial fit the profile of athletic servicemembers. Recognizing the demographic profile of our patients there have been few studies evaluating younger patients’ response to PVI. PVI techniques have been suggested to have better outcomes in younger patients with PAF when compared to patients with PersAF in smaller studies.^10^ Chun et al. observed low complication rates and reduced recurrence rates in their younger population with PAF after catheter ablation.^11^ Similar observations were made in the study conducted by Leong Sit et al. where they also observed increased rates of being AF free in their younger population under the age of 45 years after PVI.^9^ Further prospective and larger studies are needed to evaluate the efficacy and longterm outcomes of AF catheter ablation in the young, athletic individuals, and active duty personnel and current guidelines reflect this.

Military providers prescribing rate control agents and/or AADT need to recognize that many of these agents will require medical evaluation boards to comprehensively determine a servicemembers’ future occupation and deployment eligibility. Navy medical retention standards for personnel in aviation, special warfare, special operations, submarine duty, nuclear field duty, and divers are not permitted to be on rate controlling agents or AADTs and these personnel will have medical evaluation boards for determining their occupational fitness for duty.^12^ All other navy personnel in other lower risk occupations must be free of AF for more than 2 years while off medical therapy or they risk being disqualified from service. Servicemembers in the Air Force are not permitted to deploy with symptomatic AF or AF requiring medical therapy without waivers and medical board evaluation.^13^ Army personnel require medical evaluation board and fitness for duty evaluations for symptomatic AF and when AF is not adequately controlled. Army rangers and special forces personnel are not permitted to continue with these occupations with a diagnosis of atrial fibrillation.^14^ Individual case assessments with cardiologists and medical evaluation boards take into account the AF burden, severity, comorbidities, and occupation.

Assessing the value of AF ablation from the perspective of a socialized military healthcare system and the implied fiscal impact is challenging. In a study with symptomatic AF patients who failed AADT by Chan et al., their analysis signaled AF ablation being generally cost-effective. This cost benefit was most pronounced particularly in young patients with high symptom burden as they are more likely to live longer and experience drug side effects.^15^

Ultimately, the treatment decision of rate or rhythm strategy for AF should be individualized for each servicemember with respect to their treatment goals, symptom tolerance, comorbidities, and military occupation. PVI offers an opportunity for freedom from AF and the authors of this article support catheter ablation efforts in the symptomatic and/or drug-refractory young military personnel.
